# Sources and Dynamics of Inorganic Carbon within the Upper Reaches of the Xi River Basin, Southwest China

**DOI:** 10.1371/journal.pone.0160964

**Published:** 2016-08-11

**Authors:** Junyu Zou

**Affiliations:** School of Earth Sciences and Resources, China University of Geosciences (Beijing), Beijing 100083, China; East China Normal University, CHINA

## Abstract

The carbon isotopic composition (δ^13^C) of dissolved and particulate inorganic carbon (DIC; PIC) was used to compare and analyze the origin, dynamics and evolution of inorganic carbon in two headwater tributaries of the Xi River, Southwest China. Carbonate dissolution and soil CO_2_ were regarded as the primary sources of DIC on the basis of δ^13^C_DIC_ values which varied along the Nanpan and Beipan Rivers, from −13.9‰ to 8.1‰. Spatial trends in DIC differed between the two rivers (i.e., the tributaries), in part because factors controlling pCO_2_, which strongly affected carbonate dissolution, differed between the two river basins. Transport of soil CO_2_ and organic carbon through hydrologic conduits predominately controlled the levels of pCO_2_ in the Nanpan River. However, pCO_2_ along the upper reaches of the Nanpan River also was controlled by the extent of urbanization and industrialization relative to agriculture. DIC concentrations in the highly urbanized upper reaches of the Nanpan River were typical higher than in other carbonate-dominated areas of the upper Xi River. Within the Beipan River, the oxidation of organic carbon is the primary process that maintains pCO_2_ levels. The pCO_2_ within the Beipan River was more affected by sulfuric acid from coal industries, inputs from a scenic spot, and groundwater than along the Nanpan River. With regards to PIC, the contents and δ^13^C values in the Nanpan River were generally lower than those in the Beipan River, indicating that chemical and physical weathering contributes more marine carbonate detritus to the PIC along the Beipan River. The CO_2_ evasion flux from the Nanpan River was higher than that in the Beipan River, and generally higher than along the middle and lower reaches of the Xi River, demonstrating that the Nanpan River is an important net source of atmospheric CO_2_ in Southwest China.

## 1 Introduction

During the past two decades there has been increasing interest in biogeochemical processing of dissolved inorganic carbon (DIC) in freshwater riverine ecosystems at the global, regional, and local scale [1–2]. At the global scale, interest primarily stems from recent concerns over increasing atmospheric carbon dioxide (CO_2_) concentrations, and its potential role in changing global climates. More specifically, streams and rivers represent the primary conduit through which carbon (C) is transported from the terrestrial to the marine environment, approximately 50% of which reaches the world’s oceans in the form of inorganic carbon (about 0.51 Pg (10^15^ g) annually) [3]. In addition, recent studies have shown that the evasion of inorganic carbon from river systems, primarily occurring as aqueous CO_2_ and expressed as pCO_2,_ is an important component of the atmospheric carbon budget, and appears to outweigh their spatially limited surface area [1, 4–11]. In fact, the evasion of CO_2_ from rivers may be as high as 1.8 Pg C per year, which accounts for 43% of the C degassing flux from inland waters [12]. Rivers are also becoming increasingly recognized for their ability to store and process C [13] through such processes as the precipitation and dissolution of carbonate (and silicate) minerals, biotic respiration, and photosynthesis [14–16]. At the local to regional scale, inorganic carbon is an important factor controlling the buffering of stream waters against changes in pH, and therefore, the speciation and solubility of dissolved constituents (e.g., trace metals) [17] as well as the kinetics of chemical reactions within the water column. As such, it influences the geochemical nature of the aquatic environment. In light of the above, there is a clear need to document the source, processing, and fluxes of dissolved inorganic carbon (both longitudinally along the channel and by means of evasion) within river systems.

The aqueous CO_2_ in rivers is usually derived from (1) soil CO_2_ formed by the mineralization/decomposition of terrestrial organic matter and terrestrial root respiration (allochthonous) via soil/groundwater, (2) CO_2_ emissions from in situ degradation processes, and (3) CO_2_ released during the precipitation of carbonates (autochthonous) [6, 18–19]. Accordingly, rivers with various geochemical characteristics and anthropogenic activities show large spatial heterogeneities in pCO_2_ and, thus, CO_2_ evasion fluxes [19–21]. Moreover, pCO_2_ has a strong influence on the process of carbonate dissolution and subsequently the formation of DIC, which then controls inorganic carbon cycling between different carbon pools [22].

Recently, Liu et al. [23–24] questioned the traditional point of view and argued that the atmospheric CO_2_ sink associated with carbonate weathering is more significant in controlling both short-term and long-term climate changes than silicate weathering. Regardless of the role that carbonate weathering plays in controlling climate change, these previous studies have demonstrated its significant role in buffering atmospheric CO_2_ throughout Earth’s evolution and history [23–26]. Carbonate rock weathering within the Nanpan and Beipan Rivers—two headwater tributaries of Xi River—have recently drawn attention. Xu and Liu [27] investigated the major element and strontium (Sr) isotope geochemistry of water in the upper Xi River. They found that with one exception, carbonate rock weathering dominated the chemistry of major ions in the upper Xi River. The exception was for the upper reaches of the Nanpan River where the weathering of silicate minerals was also obvious. Li et al. [28] used carbon isotopic composition and major ion data from river and spring waters to confirm that sulfuric acid acted as an agent of carbonate weathering in the Beipan River and highlighted its role in combination with atmospheric CO_2_ on controlling carbonate weathering rates. Although several articles have reported on seasonal variations in pCO_2_ as well as the DIC contents and isotopic compositions in the Xi River [18, 28–31], spatial variations in inorganic carbon isotopes and the dynamics of pCO_2_ (including CO_2_ outgassing) are not well known within the upper reaches of Xi Basin. Headwater basins usually emit more CO_2_ because of higher CO_2_ partial pressures, water turbulence and wind/flow velocity [32]. In addition, the impacts of anthropogenic activities on DIC needs to be urgently documented to better understand their influence on chemical weathering processes and the carbon cycle within headwater tributaries of the Xi River.

In this study, the main objectives are to identify the sources of inorganic carbon, to better understand carbon and pCO_2_ dynamics including the factors that influence these dynamics along the channels, and to estimate the fluxes of CO_2_ outgassing along the Nanpan and Beipan Rivers.

## 2 Geographic and Hydrologic Settings

**Fig 1:**
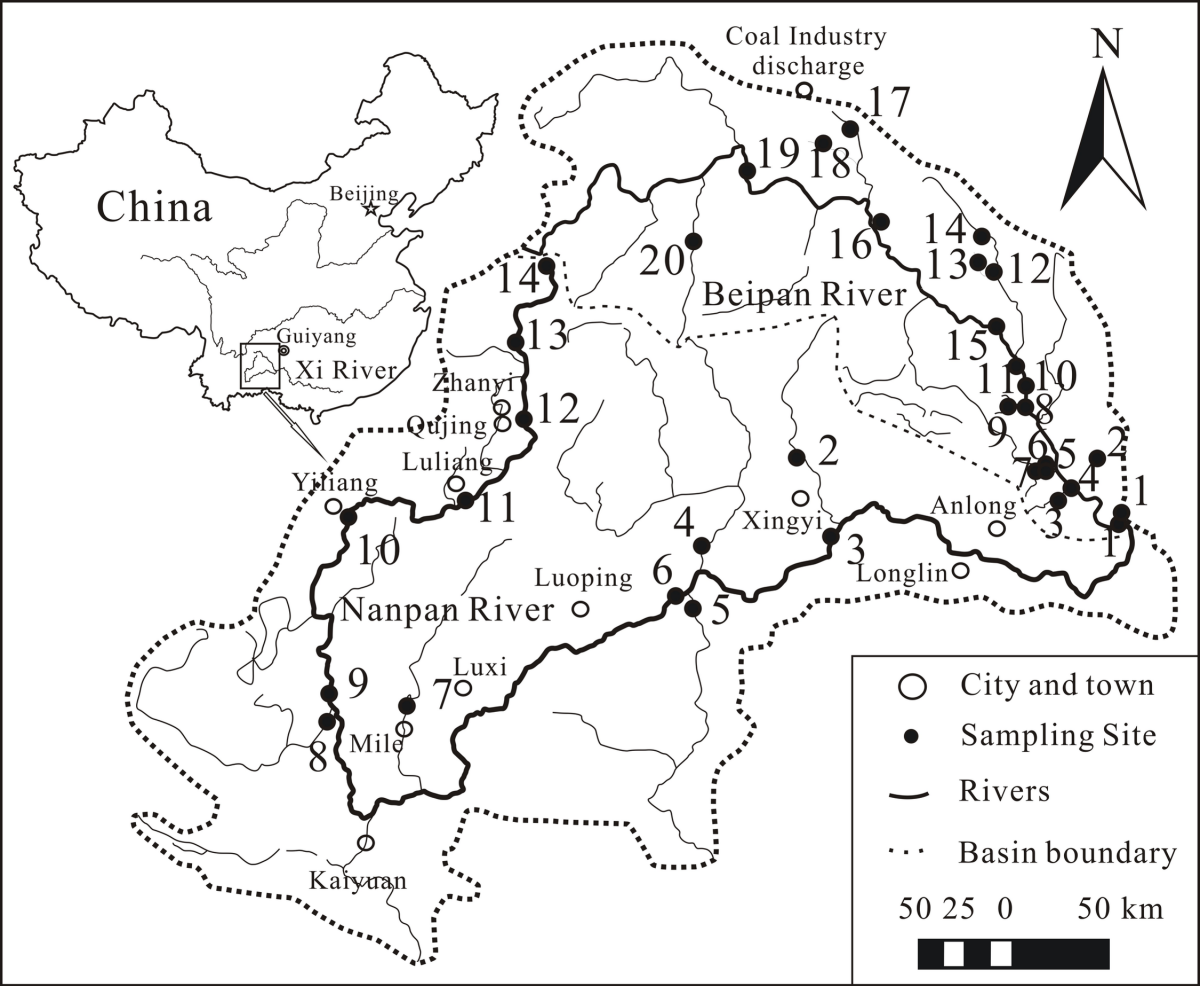
Map showing the location of sampling sites along the Nanpan and Beipan Rivers. Map based on the Geospatial Data Cloud (public domain http://www.gscloud.cn/search).

The Xi River (which drains into the mainstream of the Pearl River; Fig 1) is characterized by a distinct dry-wet subtropical climate. Average annual rainfall over several years is between 800 and 1200 mm [27]; the occurrence of a seasonal monsoon contributes to high precipitation during summer and low precipitation during winter. The precipitation during the rainy period (June to September) accounts for about 80% of the total annual precipitation. The mean annual temperature within the Xi River basin ranges between 14 and 22^○^C. The Nanpan and Beipan Rivers are headwater tributaries to the upper reaches of the Xi River. The Nanpan River exhibits a total length of 914 km, and possesses a drainage area of 56,880 km^2^; annual water discharge at its mouth is 242 × 10^8^ m^3^/yr. The Beipan River is the largest tributary of the Nanpan River, possesses a total length of 444 km, and exhibits a drainage area of 26,590 km^2^ with a maximum altitude of 1,932 m. Its annual water discharge is 143 × 10^8^ m^3^/yr. The upper reaches of the Nanpan River are underlain by detrital sedimentary and magmatic rocks. Permian and Triassic carbonate rocks are common along the lower reaches [27]. The carbonate rock stratum encompasses 55.5% of the catchment area. In contrast, Permian and Triassic carbonate rocks and coal-bearing formations dominated the Beipan River basin, covering 74.1% of the catchment area. The upper reaches of the Nanpan River flow through cities characterized by advanced industry, agriculture and sewage discharge. Water pollution is severe [33]. The Beipan River is burdened by discharged waste water and industrial sewage from numerous upstream coal mining industries in the city of Liupanshui and in southwestern areas of Guizhou Province. Water pollution and environmental problems are significant along the Beipan River as well [34].

## 3 Sample Collection, Laboratory Analysis and Methods

Fourteen (14) and 20 water samples were collected from the mainstreams and tributaries of the Nanpan and Beipan Rivers, respectively during high-flow in July, 2014 (Fig 1). The sampling conducted for this study was carried out in areas where specific permission for sampling was not required. Moreover, field studies did not involve work with endangered or protected species

All of the water samples were collected in 10 L low-density polyethylene (LDPE) containers at 0.5 m below the water surface in the center of the main channel or its tributaries. Temperature, pH and dissolved oxygen (DO) of the water samples were measured at the sampling sites using a portable multi-parameter water quality meter (WTW Germany multi3410). The HCO_3_^−^ concentration was determined by 0.025 M HCl titration within 12 h of sampling. Alkalinity, as investigated here, refers to the buffering capacity of the carbonate system in water and can be expressed for karstic freshwaters by the following equation [29]: Alk = [HCO_3_^-^] + 2[CO_3_^2-^] + [OH^-^]–[H^+^]. Alkalinity was determined within 12 h of sampling using a titration method involving 0.01 M HCl. For each sample, three replicates were analyzed by titration to determine analytical error (1 σ), which was <3%. Samples were filtered through 0.45 μm cellulose-acetate filter paper and preserved with HgCl_2_ to prevent biological activity in 125 ml polyethylene bottles for the carbon isotopic composition of DIC [28]. The particulate solid was then divided into two subsamples; the carbonate was removed from one subsample using HCl prior to the analysis of particulate organic carbon (POC). The other subsample was not pre-processed and was used for the analysis of total carbon (TC = POC + PIC). The difference between the concentrations determined for POC and TC was assumed to be the concentration of particulate inorganic carbon (PIC). Eighty-five percent H_3_PO_4_ was added to the bottles with water and particulate solid to produce CO_2_ gas in the headspace for the determination of δ^13^C [35]. The contents of PIC were determined by reference to a sulfanilamide standard, which consisted of N (16.25%) and C (41.81%), using an Elementar Vario MICRO cube. Replicate analysis indicated a precision of < ± 0.5%.

The water samples were added to the bottle, which had been pre-purged with 99.99% high-purity helium gas for 60 min (the modified pre-purging) [35]. Then 85% H_3_PO_4_ was added, and the mixture was heated in a 60°C water bath for 60 min (the optimal reaction conditions). The δ^13^C_DIC_ value of the CO_2_ gas in the headspace was then determined. For the measurement of PIC, the particulate samples were added to the bottle before they were purged with 99.99% high-purity helium [35]. Carbon isotopic analysis of the DIC and PIC were determined using a GasBench online high-precision gas headspace sample coupled with a MAT-253 isotope ratio mass spectrometer (Thermo Fisher Scientific, Bremen, Germany; with a precision of 0.03‰; [35]). The carbon isotopic composition of DIC and PIC were reported using the δ notation relative to PDB in per mil, where δ^13^C (‰) = [(R_sample_ − R_PDB_) / R_PDB_] × 1000.

Aqueous inorganic carbon species include CO_2_, H_2_CO_3_, HCO_3_^−^ and CO_3_^2−^. Given the range of measured pH values, bicarbonate (HCO_3_^−^) was the dominant DIC species. Therefore, the concentrations of DIC are assumed to be equal to HCO_3_^−^ in this article [36]. The pCO_2_ values were determined based on measured alkalinity, pH and water temperature using the CO2SYS program [29], where the constants K_1_, K_2_ are dependent on the temperature from Millero [37]. The calcite saturation indexes (SIc) were calculated using the thermodynamic constants at a given temperature [22, 38].

## 4 Results

### 4.1 δ^13^C of inorganic carbon

In this study, with one exception (−13.9‰ at NPJ-9), the Nanpan River exhibited a small range of δ^13^C_DIC_ values (from −11.4‰ to −9.5‰); the mean value was −10.6‰. The δ^13^C_DIC_ values in the Beipan River varied from −12.3‰ to −8.1‰, and exhibited a mean value of −10.3‰.The δ^13^C values of PIC ranged from −9.1‰ to −1.5‰ and from −3.1‰ to −0.8‰ for the Nanpan and Beipan Rivers, respectively. The mean value of δ^13^C for the Beipan River (−2.0‰) was generally higher than that for the Nanpan River (−4.3‰).

### 4.2 pCO_2_ and calcite saturation index (SIc)

**Fig 2:**
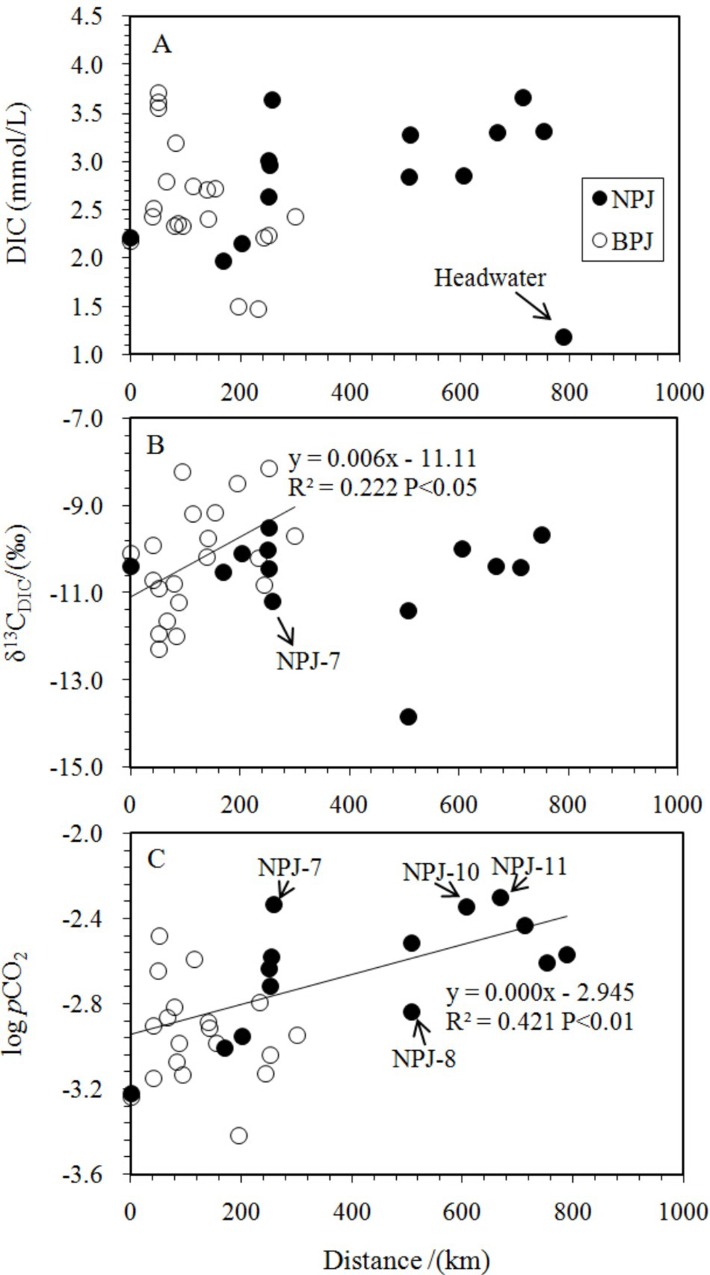
**Spatial distribution in DIC (a), δ^13^C_DIC_ (b), and logpCO_2_(c) along the studied rivers; NPJ: Nanpan River samples, BPJ: Beipan River samples.** Distance refers to distance to the outlet of the basin (S1 Table). The legends in the following figures are the same to the Fig 2.

The DIC contents of the Nanpan River ranged from 1.18 mmol/l to 3.65 mmol/l with an average of 2.78 mmol/l. The concentration of DIC within the Beipan River Basin varied from 1.47 mmol/l to 3.61 mmol/l, with an average of 2.51 mmol/l. The DIC of the two rivers showed opposite spatial (longitudinal) trends (Fig 2). Similar variations in logpCO_2_ to DIC concentrations of the two rivers are shown in Fig 2. The pCO_2_ values calculated for the Nanpan and Beipan Rivers ranged from 599 μatm to 5006 μatm and 379 μatm to 3296 μatm, respectively. These pCO_2_ values were generally higher than 380 μatm (the value of atmospheric pCO_2_; [18]). At the calculated pCO_2_ conditions, with one exception from the headwater regions of the Nanpan River, most water samples had SIc values greater than zero, indicating the waters in both rivers were oversaturated relative to calcite. The concentrations of PIC for the Nanpan River ranged from 0.19 mg/l to 4.41 mg/l, in contrast to 0.01 mg/l to 6.81 mg/l for the Beipan River.

## 5 Discussion

### 5.1 Carbon isotopic composition of dissolved inorganic carbon in the rivers

Both the range and the average values of δ^13^C_DIC_ for the Beipan River were lower than that for the Nanpan River. These lower values in the Beipan River are in spite of a larger proportion of carbonate rock coverage and a lower dilution effect in response to lower precipitation during the wet season. It is possible that heavy rainfall events in the Nanpan River catchment facilitate reactions between minerals and soil CO_2_. Minor carbonate minerals eroded from silicate rocks also could play an important role in the formation of DIC [24]. In fact, this may be one of the reasons for a significant amount of ^12^C-enriched DIC/CO_2_ from soil and may contribute to the chemical weathering of carbonate and silicate rock characterized by the low δ^13^C values found in DIC.

**Fig 3:**
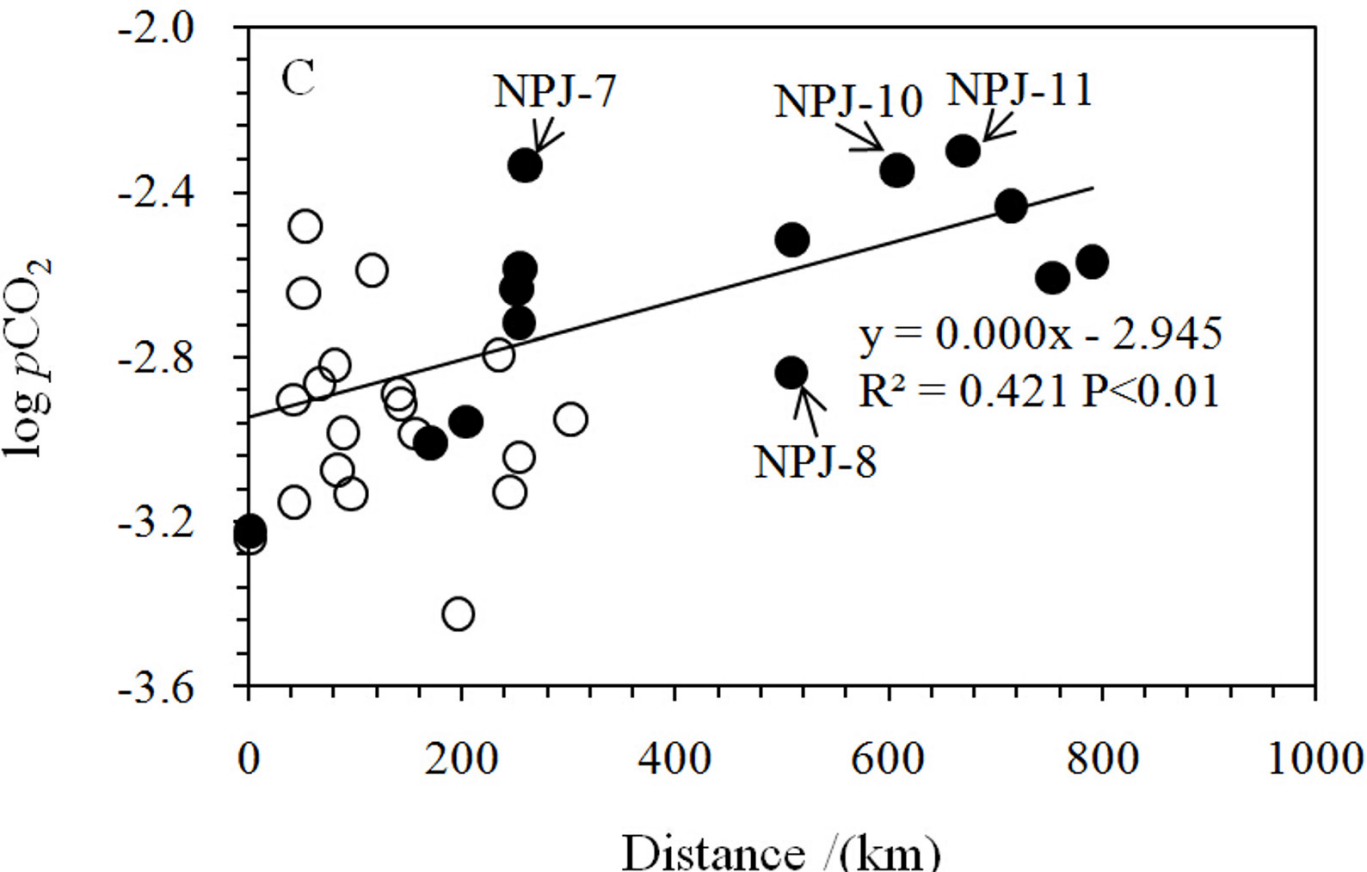
Correlation between DIC content and δ^13^C_DIC_. The trend line applies to data from the BPJ.

**Fig 4:**
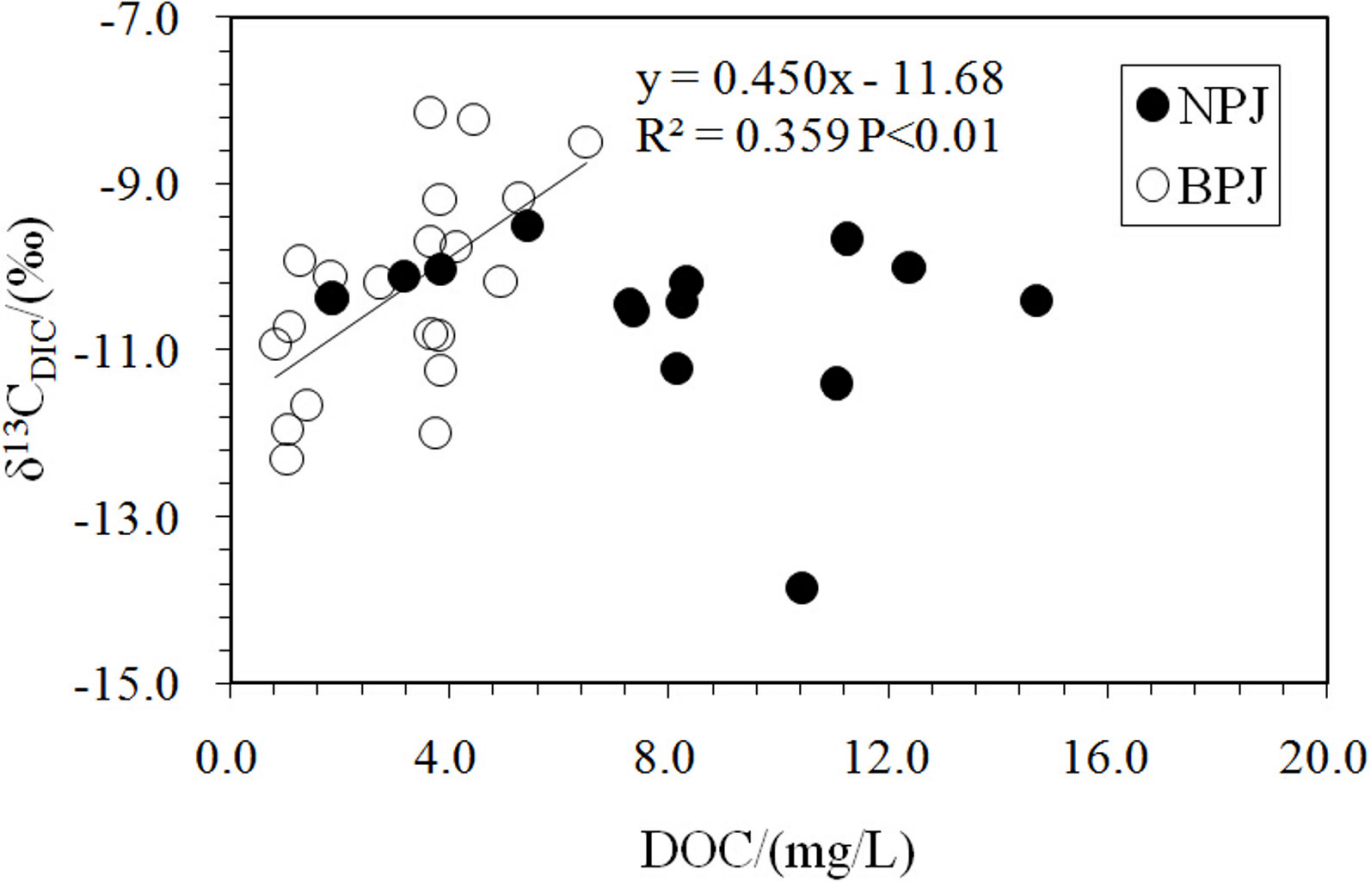
Correlation between δ^13^C_DIC_ and DOC content. The trend line applies only to BPJ; no statistically significant relationship exists for NPJ. DOC data cited from Zou (in review).

A weak inverse correlation between DIC concentration and carbon isotopic composition was observed along the Beipan River (Fig 3; R^2^ = 0.350, P < 0.01). This trend may be due to mixing (soil CO_2_ flushing, CO_2_ from in situ biodegradation and CO_2_ consumption during photosynthetic activity), as was found for the Rhone and Houzhai Rivers [39, 22]. A positive correlation between δ^13^C_DIC_ and DOC, as shown in Fig 4 (R^2^ = 0.359; P < 0.01), implies that the oxidation of organic matter was a major source of DIC. In marked contrast, the δ^13^C_DIC_ values for the Nanpan River were around −11‰ (Figs 2 and 4), a typical value observed where DIC is derived from the dissolution of carbonate minerals by carbonic acid in soils in southwest China [22].

**Fig 5:**
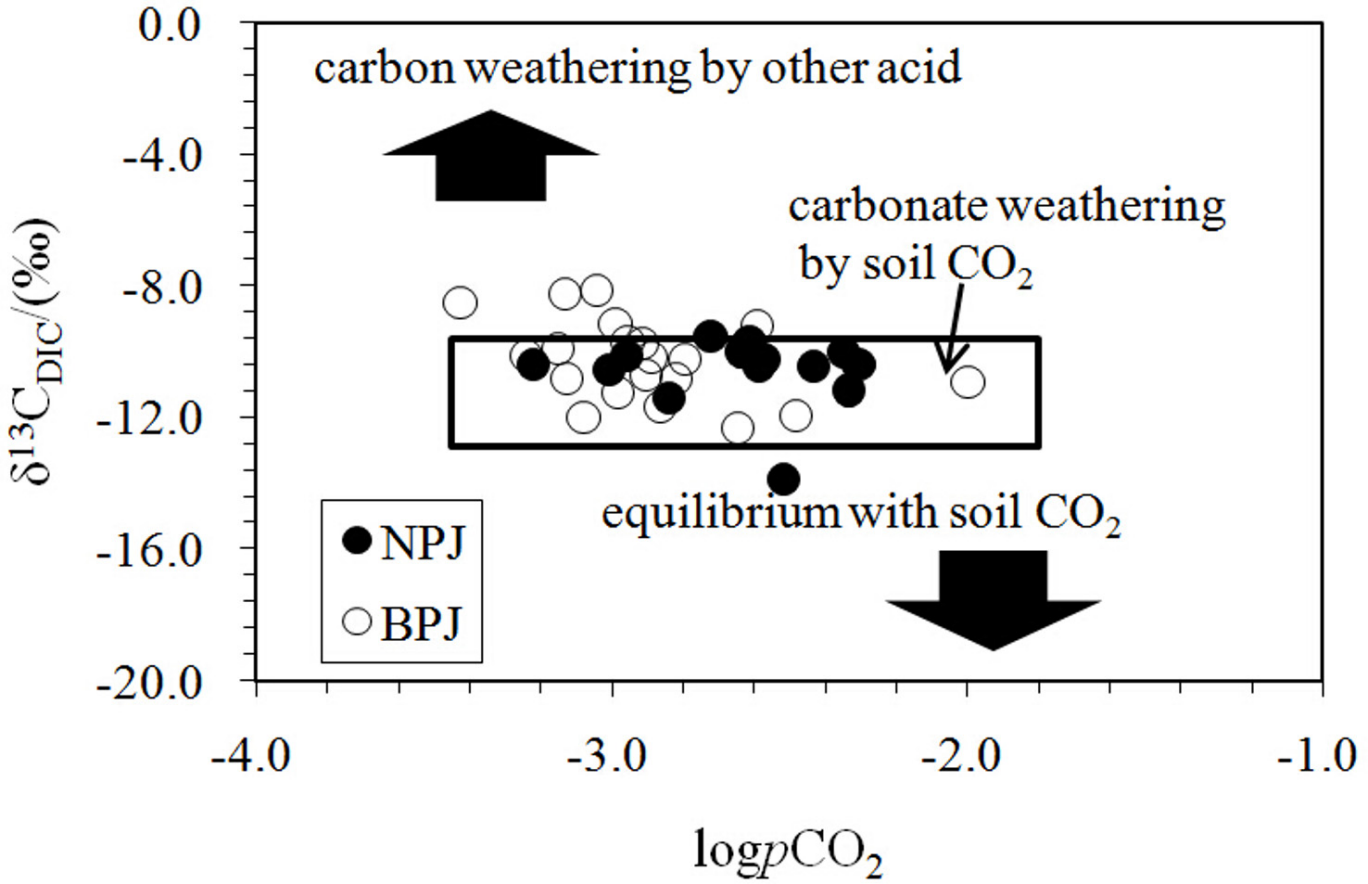
Plot showing changes in δ^13^C_DIC_ as a function of logpCO_2_. The range of carbon isotopic and logpCO_2_ values shown by the box for carbonate weathering was obtained from a previous investigation within the Houzhai catchment, southwest China [22]. The Houzhai catchment has a similar type of plants and cultivation to the upper reaches of Xi River.

Previous studies showed that DIC in catchments in southwest China may have two primary sources, soil CO_2_ and the dissolution of carbonate minerals [22, 28–29]. The relative proportion of C_3_ over C_4_ plants and the rate of CO_2_ diffusion dominate the isotopic composition of soil CO_2_ which is derived from heterotrophic oxidation of soil organic matter and respiration from plant roots [18]. Both of these processes produce soil CO_2,_ and occur with negligible isotopic fractionation between the organic matter substrate and the CO_2_ produced [40]. Li et al. [28] reported that the δ^13^C_POC_ values are close to −25‰ in surface waters in the upper Xi River. Diffusion of CO_2_ has been shown to cause an isotopic enrichment of 4.4‰ [41]. Accordingly, the δ^13^C of soil CO_2_ is approximately −21‰. Clark and Fritz [38] suggested that karst areas characterized by the rapid infiltration of surface waters to the water table could be considered as closed systems. Therefore, δ^13^C_DIC_ values that result from the dissolution of carbonate rock (0‰) by soil CO_2_ should be around −11‰±2‰ [22]. These results suggest that carbonate weathering by carbonic acid originated from soil CO_2_ is important in both rivers (Fig 5).

In comparison to other river systems, the δ^13^C values of the samples are generally lower than that of the Indus, Colorado and St. Lawrence Rivers which frequently exchange C with atmospheric CO_2_ due to the presence of lakes and dams [5, 41–44]. The δ^13^C values also are lower than those of Ganges-Brahmaputra and Lesser Antilles rivers which were affected by metamorphic and magmatic CO_2_, respectively [45–46], and the Rhone and Yangtze Rivers which are influenced by sulfuric acid [39, 47]. The δ^13^C values are higher than that of the upper reaches of the Ottawa River basin characterized by soil respiration and silicate weathering [48], the Lagan River affected by anthropogenic inputs [4], and groundwaters in southwest China which were more affected by the degradation of organic matter in the soil [36]. The δ^13^C values were similar to the Brahmaputra basin [49], the Wu River [50] and the Houzhai catchment [22]. When combined, the cited results indicate that the observed variations of δ^13^C_DIC_ values may be influenced by multiple factors, including soil CO_2_ produced by root respiration and microbiologic degradation, dissolution of carbonate rock, isotopic exchange with the atmosphere by degassing of CO_2_, the involvement of sulfuric acid derived from the dissolution of evaporates, the oxidation of sulfuric minerals and coal-containing strata, and various types of anthropogenic inputs (coal mining, sewage etc.). Although photosynthetic uptake of DIC by aquatic organisms has been shown to be an important component of the carbon budget [26], photosynthetic effects may be insignificant because of a dynamic karstic hydrological system in the case of the upper reaches of the Xi River.

### 5.2 pCO_2_ dynamics

The pCO_2_ in rivers is regulated by both internal carbon dynamics and external biogeochemical processes. These processes consist of four major factors [18–19, 51]: (1) transport of soil CO_2_ produced by the decomposition of organic matter and plant respiration by means of baseflow and interflow, (2) in situ organism respiration and degradation of organic carbon within the water column, (3) photosynthetic activity by aquatic plants, and (4) CO_2_ evasion from water to air. The former two processes enhance CO_2_ levels, while the last two can be responsible for CO_2_ decreases.

**Fig 6:**
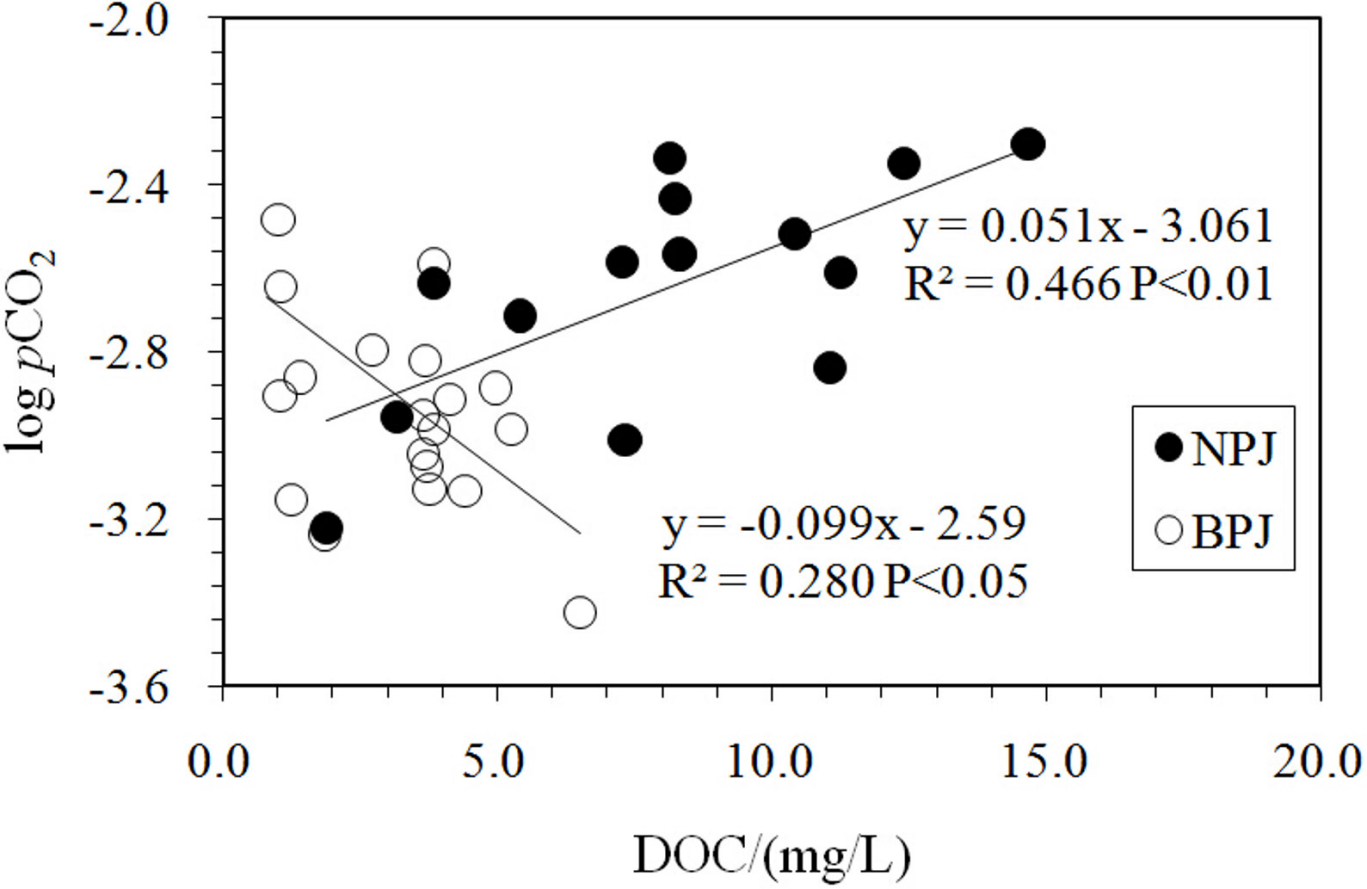
Variation of logPCO_2_ with dissolved oxygen (DO). Inverse relations were observed for both the Nanpan and Beipan Rivers. logPCO_2_ represents the logarithmic value of PCO_2_.

During the wet season, when 80% of the total annual precipitation occurs, higher temperatures and low retention times of soil waters, combined with active bacterial activities, leads to the production and flushing of a significant amount of soil CO_2_ [48, 52]. The enhanced dissolved soil CO_2_ is transported via hydrologic conduits (baseflow and interflow) to the rivers. Along its pathway, bio-degradation may occur. Variations in CO_2_ transport and degradation result in spatial variations in pCO_2_. In return, aqueous pCO_2_ values are also diluted by intense rainfall, surface runoff and discharge [18, 53]. Moreover, dams and the associated “artificial lakes” may lead to lower suspended matter and turbidity, higher residence time, thermal stratification and alterations in light conditions within the river waters [54]. These changes in the aquatic environment may then lead to biogenic CO_2_ uptake (photosynthesis) and the release (respiration) within the water column, both of which can adjust aqueous pCO_2_ levels within the reservoirs as well as along other low-flow and low-turbidity reaches [5, 55]. The pCO_2_ along the Nanpan and Beipan Rivers was negatively related to SIc (R^2^ = 0.46, P = 0.01 and R^2^ = 0.35, P < 0.01) and positively correlated to DIC contents (R^2^ = 0.68, P < 0.001 and R^2^ = 0.71, P < 0.001), indicating a strong influence of pCO_2_ on carbonate dissolution and an increase in the formation of DIC within headwaters of the Xi River [22]. In addition, the effects of oxygen consumption are apparent in Fig 6. As mentioned above, the difference between the average concentrations of DIC in the two rivers was 0.27 mmol/l, while the pCO_2_ values of the Nanpan River (2644 μatm) were twice as high as the Beipan River (1287 μatm). These trends suggest that there are different controlling factors on pCO_2_ between the two rivers.

### 5.3 Variation in pCO_2_ and the controlling factors

**Fig 7:**
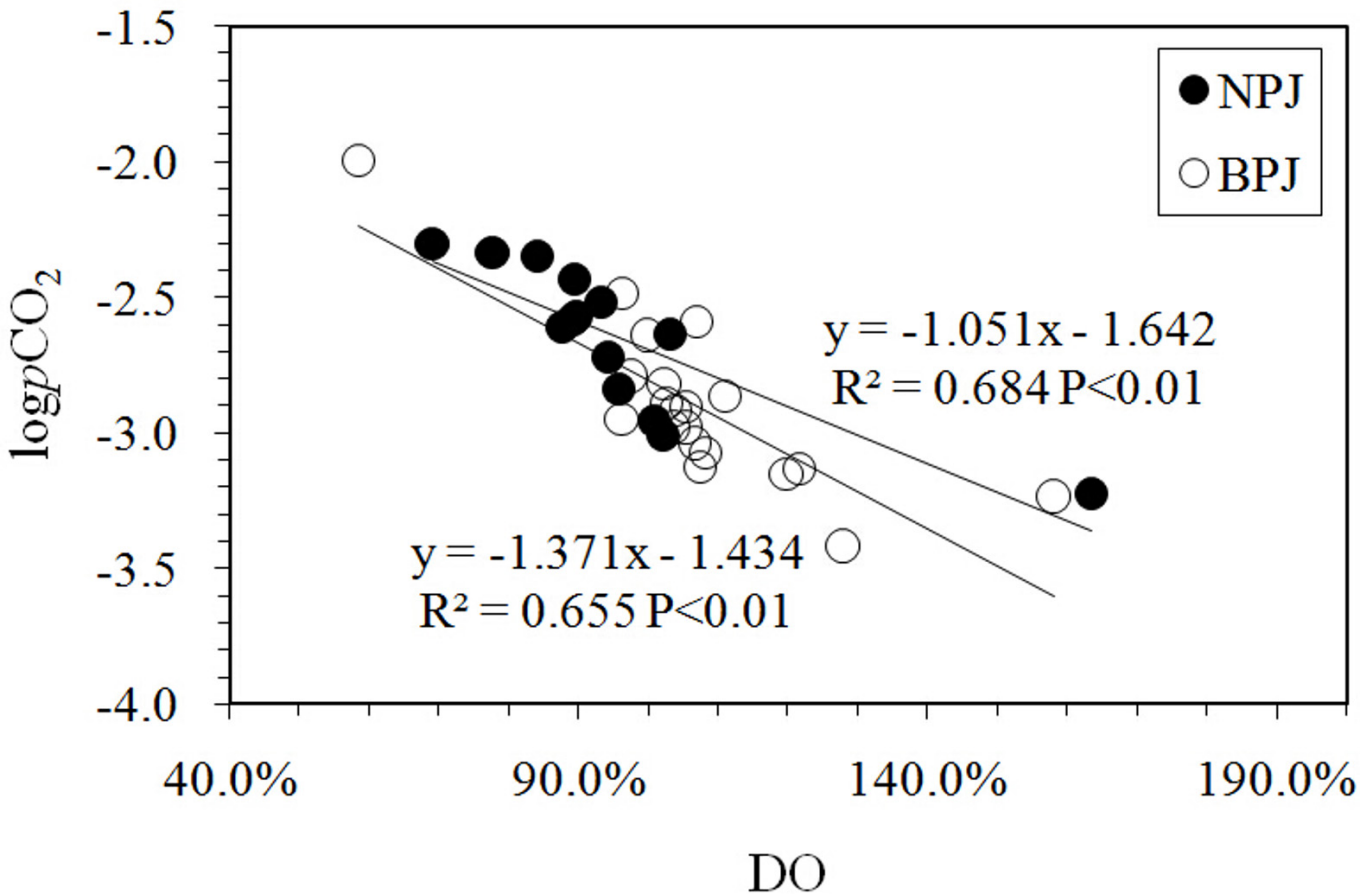
Variations in logPCO_2_ with DOC. Contrasting positive and inverse correlations were observed for the Nanpan and Beipan Rivers, respectively. See Fig 6 for logPCO_2_ data.

As shown in Fig 6, the logpCO_2_ values were significantly negatively correlated with DO (R^2^ = 0.655, P < 0.01), suggesting that oxygen consumption processes were dominant along the Nanpan River (e.g., respiration, bio-degradation, oxidation). However, the logpCO_2_ values exhibit a positive correlation with DOC contents (R^2^ = 0.48, P < 0.01) (Fig 7). These results demonstrate that the degradation of organic matter was restricted, and the transport of soil CO_2_ and organic carbon through hydrologic conduits (baseflow and interflow) were a primary control on pCO_2_ levels. Moreover, they reflect complicated carbon dynamics and biogeochemical processes that occur during the wet season [18].

Spatial variations in pCO_2_ values were divided in two parts by the Huaxi River sampling sites (NPJ-8) as shown in Fig 2C. Sample NPJ-14 was collected in the headwaters of the Nanpan River in an area located away from anthropogenic activities. The water was characterized by the lowest observed pH and SIc (−0.9), the lowest DIC concentration and turbidity, and a high pCO_2_, all of which indicate that the water was under saturated with respect to calcite. In other words, the amount of DIC from calcite was relatively low, and a significant amount of soil CO_2_ was dissolved in the water resulting in higher pCO_2_ values and the lowest pH The upper reaches of the Nanpan River show a parabolic trend in pCO_2_ along the channel and are characterized by high DOC (8.24–14.69 mg/l) and DIC contents (2.84–3.65 mmol/l) and lower DO (69.2%–95.6%). The maximum values of pCO_2_ in the upper reaches of the Nanpan River were found at NPJ-11 and NPJ-10. Organic pollutants from the Qujing and Luliang, industrially developed cities [56], could become the major sources of CO_2_. The pCO_2_ in the lower reaches of the Nanpan River exhibit a decreasing downstream trend which indicates that respiration was becoming limited and photosynthesis was relatively significant. Therein, the highest pCO_2_ from the Dixian River (NPJ-7), a tributary to the Nanpan River, may result from enhanced respiration induced by human activities (e.g., rural cultivation and reservoir construction), which exhibited the lowest δ^13^C_DIC_ value (−11.2‰) and DO (77.8%) values among the values of the lower reaches (Fig 2B). Moreover, in general the concentrations of Cl^−^ (0.39–0.56 mmol/l) in the upper Nanpan River were much higher than the lower reaches (0.06–0.27 mmol/l) (S1 Table), reflecting the heavy discharge of cities and towns.

Aitkenhead and Mcdowell [57] found that vegetation types and soil properties are the key to soil CO_2_ preservation and DOC fluxes. Coniferous forests have lower pCO_2_ values than broadleaf forests [58]. Fertilization involving N, P, C, Fe, Zn, and Si increases organic matter storage/burial by aquatic organisms and thus decreases the return of CO_2_ to the atmosphere [26]. Cropland is widely distributed in the upstream areas, while broadleaf deciduous forest mixed with cropland is common in the downstream portions of the basin. Although cropland covers a large amount of area in the upper reaches of the Nanpan River, pCO_2_ values were elevated along the lower reaches of the river, suggesting that urbanization and industrialization contribute more pCO_2_ than do agricultural activities and enhance alkalinity [59–60].

In contrast to the Nanpan River, logpCO_2_ along the Beipan River is negatively correlated with DOC (R^2^ = 0.27, P < 0.05) (Fig 7), suggesting that the oxidation of DOC was an important source of pCO_2_. Lower terrestrial organic carbon input might be the main reason that pCO_2_ levels are lower than for the Nanpan River [55]. In addition, water samples in the upper reaches of the Beipan River had heavier δ^13^C_DIC_ values and lower pCO_2_ values (Fig 2), in spite of the high downstream variability in pCO_2_ values along the Beipan River. The involvement of sulfuric acid in carbonate weathering is most likely responsible for the positive shift of δ^13^C_DIC_ ([28]; Fig 5). The competition between carbonic acid and sulfuric acid for the pCO_2_ is obvious. The sample (BPJ-16) collected at the Zangke River scenic spot possessed the lowest pCO_2_ value and exhibited a relatively high δ^13^C_DIC_ value. The involvement of sulfuric acid cannot explain this observed relationship because the reach also exhibited higher pH and DO values and lower DIC concentrations. The discharge of human waste and the input of nutrients, combined with other human activities at the scenic spot have resulted in eutrophication. Eutrophication can either increase or decrease pCO_2_, but which it does depends on the balance between the amount of DOM oxidation that occurs (a process that increases pCO_2_) and the degree of primary production that is enhanced as a result of nutrients (i.e., photosynthesis which decreases pCO_2_). Aquatic photosynthesis that draws down pCO_2_ and consumes DIC is presumably occurring, which leads to a lower DIC content, and higher pH and DO values. However, we should note the complexity inherent in the system with regards to the controls on pCO_2_. Primary production leads to a decrease in pCO_2_ values and higher δ^13^C_DIC_ values. Degassing of CO_2_ tends to increase δ^13^C_DIC_ within the remaining DIC and decrease it within pCO_2;_ such degassing will increase the δ^13^C_DIC_ by about 0.5‰, which is inconsistent with the lowest pCO_2_ value measured at the scenic spot. Thus, primary production appears to dominate at this site. However, this may not be the case everywhere. The waters of the Luofan River (BPJ-5; BPJ-7) along the lower reaches of the Beipan River exhibit higher DIC content and pCO_2_ values, which can be interpreted by the mixing of river waters with groundwater (BPJ-6), the latter characterized by the highest pCO_2_ and DIC. The channel near the outlet, which is affected by frequent human activities, is wide, and characterized by slow moving, clear water. It is similar to a scenic spot along the Zangke River where low pCO_2_ and higher DO near its outlet could be explained by the production of CO_2_ by the oxidation of organic carbon, a process that maintained the pCO_2_ levels. It also was affected by anthropogenic activities.

It is thus clear that industrialization and urbanization help establish the observed levels of pCO_2_ and promote the formation of the measured DIC content along the upper reaches of the Nanpan River characterized by silicate bedrock. Human activities can also explain why the rivers had equal DIC contents, while distinct pCO_2_ levels.

Previous studies have found that the pCO_2_ is often elevated in small, low-order, headwater channels and decreases downstream. Elevated upstream pCO_2_ values are often attributed to the influx of soil waters highly charged with CO_2_ [8, 52, 61–62]. As noted above, a similar downstream trend was observed for the Nanpan River. However, it is important to recognize that the observed downstream geographical pattern in pCO_2_ was related to multiple factors, each of which influenced pCO_2_ along different reaches (segments) of the river. Not only did the controls on pCO_2_ vary along individual reaches of the studied rivers, but between the two river basins. Along the Beipan River, for example, the pCO_2_ is highly variable reflecting both natural and anthropogenic influences.

The complexity observed in the controlling factors of pCO_2_ along and between rivers is significant in that it makes it difficult to assess the sources of DIC and to extrapolate and predict CO_2_ concentrations in rivers on a regional scale without detailed information on individual river basins. The required data may include the input of terrestrial organic matter, the ratio of groundwater discharge to surface runoff, chlorophyll α, and primary production (photosynthesis) and community respiration etc. [13]

### 5.4 Particulate inorganic carbon (PIC) dynamics and carbon isotope composition

Carbonate in soil is often produced by the deposition of Ca^2+^ and HCO_3_^−^ under nonequilibrium conditions and from the deposition of carbonate containing dust. These ions are usually derived from the weathering of silicate and carbonate rocks [23]. Waters in the Nanpan and Beipan Rivers are generally oversaturated with respected to calcite (Sic > 0). The δ^13^C of authigenic calcite is taken as −12‰, or the composition computed for precipitation at equilibrium within the water column [39]. The average δ^13^C value of marine Paleozoic to Tertiary carbonate rocks is about 0.5‰ [63]. These δ^13^C values of PIC suggest that the contribution of marine carbonate detritus to the PIC in the Beipan River was greater than to the Nanpan River. In addition, the mean PIC content within waters of the Beipan River (2.76 mg/l) was higher than that in the Nanpan River (2.03 mg/l). These results imply that there is intense physical and chemical erosion of the carbonate rocks in the Beipan River Basin.

### 5.5 CO_2_ evasion to the atmosphere

In general, the gas transfer coefficient (D / Z) is the predominant factor controlling the CO_2_ evasion flux from a point source [64]. Aqueous CO_2_ can evade unidirectionally into the atmosphere along the water-to-air interface as a result of higher aqueous pCO_2_ values than in the air [4]. In this case, the CO_2_ evasion flux can be relatively low [32]. In terms of the whole catchment, the average pCO_2_ values can be used to assess the degree of evasion [18].

The flux of CO_2_ (F) across the water-to air interface can be calculated on the basis of a theoretical diffusion model [18–19] expressed as:

F = D / z× (pCO_2water_ − pCO_2air_) / K_h_

where D / Z is the gas exchange coefficient (D is the diffusion coefficient of CO_2_ in the river; z is the thickness of boundary layer; [18]), which is related to river runoff, turbidity, flow velocity, water depth and wind speed, etc. and may vary from 4−115 cm/h [1, 19, 39]. The quantity pCO_2water_−pCO_2air_ is the difference in pCO_2_ between the overlying air and the average value of the water; while K_h_ is Henry’s constant, a value taken as 22.4 μatm/(mol/m^3^) [18], close to the value of 29.4 μatm/(mol/m^3^) at 25°C [65]. The pCO_2air_ value is about 380 μatm. Given a mean wind speed of 1.9 m/s and hydrological features in the upper reaches of the Xi River in comparison to the lower reaches [18]; a D / Z value of 8 cm/h (1.92 m/d) can be used to estimate the lower limited of CO_2_ degassing flux [18].

As discussed by Hunt et al. [66], a significant contribution of organic acids to total alkalinity (TA) leads to an overestimation of calculated pCO_2_ with the CO2SYS program, or with any program that accounts only for the inorganic species that contribute to TA. However, the approach of calculating pCO_2_ from pH, TA and temperature is robust in freshwaters with circum-neutral to basic pH and with a TA exceeding 1000 μmol/ l, including karst rivers [67]. This is likely to be particularly true for the upper reaches of the Xi River characterized by a TA in excess of 2000 μmol/ L and that exhibits a pH around 8 (at which point HCO_3_^-^ is the dominant species driving TA and found in DIC; Table 1). Thus, organic alkalinity typically can be neglected in this research [67].

**Table 1:** The average pCO_2_ and CO_2_ evasion flux along different reaches of the Xi River during the wet season and documented for other rivers.

River	Country	Climate	pH	DIC (mmol/l)	pCO_2_ (μatm)	FCO_2_ mmol m^-2^ d^-1^	Reference
**Nanpan River**	China	Subtropic	7.9	2.78	2644 (Summer)	194 (Summer)	This study
	China	Subtropic	7.9	2.97	2365 (Summer)	170 (Summer)	[27]
**Beipan River**	China	Subtropic	8.1	2.57	1287 (Summer)	78 (Summer)	This study
	China	Subtropic	8.2	2.52	1051 (Summer)	58 (Summer)	[27]
**Hongshui River**	China	Subtropic	8.3	2.77	886 (Summer)	43 (Summer)	[68]
**Qian and Xun River**	China	Subtropic	8.1	2.06	943 (Summer)	48 (Summer)	[68]
**Xi River (downstream)**	China	Subtropic	8	1.95	1270 (Summer)	76 (Summer)	[68]
	China	Subtropic	7.7	1.56	2374 (Summer)	171 (Summer)	[18]
**Xi Rvier**	China	Subtropic	7.6	1.58	2600	190–357	[18]
**Longchuan River**	China	Subtropic	6.3–8.5	1.08–4.58	1230–2100	74–156	[19]
**Maotiao River**	China	Subtropic	7.4–9	2.6–3.02	3740	295	[54]
**Yangtze River**	China	Subtropic		1.7	1297	14.2	[71]
**Sinnamary**	French Guiana	Tropic				30–461	[72]
**Lower Mekong**	East Asia	Tropic	7.7	1.59	1090	195	[73]
**Amazon**	Brazil	Tropic			4350	189	[1]
**St. Lawrence**	Canada	Temperate	7.3	0.46	1300	78–295	[73]
**Ottawa**	Canada	Temperate	7	0.05–3	1200	81	[48]
**Mississippi**	USA	Temperate	7.9	0.54	1335	270	[73]
**Hudson**	USA	Temperate			1125	16–37	[73]
**Gäddtjärn headwater**	Sweden	Boreal	3.8–5.4	<0.1	2266	983	[64]
**Eastmain, Quebec**	Canada	Boreal		<0.1	611	16	[73]
**Auchencorth Moss**	Scotkand UK	Boreal		<0.1	25418	2.6	[74]
**Vindeln River**	Northern Sweden	Boreal		<0.1	722–24167	1	[75]

In this study, the results of the mean pCO_2_ value for the Nanpan and Beipan River were slight higher than the values calculated on the basis of the datasets from Xu and Liu [27]. For the lower reaches of the Xi River, the values reported by Yao et al. [18] were significantly higher than those calculated by Xu and Liu [68] using CO2SYS (Table 1), which may be due to differences in utilized equilibrium constants [19]. Our results demonstrated that CO_2_ evasion fluxes in the Xi River Basin could be under-estimated, resulting in little difference between the upper and the lower reaches (Table 1). The spatial trends in evasion of CO_2_ observed between the upstream rivers (i.e., the Nanpan and Beipan Rivers) and the downstream segments of the Xi River are consistent with other studies that have shown that carbon evasion fluxes tend to be higher upstream as a result of higher pCO_2_ values and increased turbulence along the water-air interface that leads to evasion (Table 1). Regionally, differences in precipitation, surface area, and net primary production between the upper and lower reaches of rivers could be key factors controlling evasion flux and flushing of CO_2_ from soil [52]. Few previous studies have documented the influence of human influences on carbon evasion fluxes along a river channel [2, 13, 18]. Here carbon evasion fluxes for the Nanpan River, impacted by human activities, are relatively high.

Given that the evasion of CO_2_ from rivers represents a significant component of the atmospheric C budget, it is essential to quantitatively determine the CO_2_ flux from river waters. Data generated in this study show that evasion was nearly three times higher along the Nanpan River than from the Beipan River. These differences presumably reflect, in part, higher CO_2_ concentrations measured for the Nanpan River and differences in the factors controlling the source and pCO_2_ values between the two river basins. In comparison to other subtropical rivers, C fluxes from the upper reaches of Xi River are similar to values obtained in other studies (Table 1). The differences observed between the Nanpan and Beipan rivers, combined with the variations shown in Table 1 for other subtropical rivers, indicate that the CO_2_ evasion from subtropical inland rivers is plagued by large uncertainties due to different climate, vegetation and soil and groundwater characteristics [69–70]. These uncertainties will make it difficult to accurately estimate the contributions of CO_2_ from subtropical rivers to the atmospheric carbon budget. Future studies should focus on the impacts of specific land use/land coverage changes and associated anthropogenic activities on the local and regional carbon cycle.

The values of CO_2_ evasion from the Nanpan and Beipan Rivers are lower than those measured for tropic rivers, but generally higher than for temperate rivers (Table 1). This is not surprising given that tropical river systems are thought to account for approximately 70% of the global riverine carbon fluxes [62]. Nonetheless, the evasion fluxes measured for the headwater streams in this study show that the evasion of C from subtropical rivers is not trivial, and, thus, it is essential to develop effective means of assessing the source of DIC, and the evasion of CO_2_ from their waters.

## 6 Conclusions

The degradation of organic matter and the dissolution of carbonate minerals in soil are the primary source of DIC. Carbonate dissolution is strongly affected by pCO_2_ in the upper reaches of Xi River. The factors controlling pCO_2_ between the two rivers differed. Urbanization and industrialization had a strong influence on the pCO_2_ and the formation of DIC in the upper reaches of the Nanpan River characterized by silicate bedrock. In contrast, the lower reaches exhibited a downstream decrease as a result of enhanced photosynthesis, and the subsurface transport of soil CO_2_ and organic carbon through baseflow and interflow. In addition, the involvement of sulfuric acid from coal related industries had a significant impact on the carbon evolution. The oxidation of organic carbon was the pump to maintain pCO_2_ levels. The δ^13^C values of PIC in the Nanpan River were generally lower than those in Beipan River, indicating that the contribution of marine carbonate detritus to the PIC in the Beipan River was greater than the Nanpan River. The average pCO_2_ value of the Nanpan River was much higher than the Beipan River, which implied that the Nanpan River exhibited a higher evasion flux than the Beipan River. The upper reaches of the Xi River emitted larger fluxes than the lower reaches, and headwater tributaries should be emphasized in the development of regional net carbon budgets.

## Supporting Information

S1 Table Sampling sites and geochemical index along the Nanpan and Beipan Rivers during the wet season. BPJ and NPJ represent the mainstream and tributary of the Beipan River and the Nanpan River; “-”: undetected—quantity of solid sample was insufficient for measurement; “*”: no data—samples were not collected in the field; Distance refers to distance to the outlet of the basin (data was measured by Arcgis). DOC datasets cited from Zou (in review). DOC concentrations were determined on an Aurora 1030W TOC Analyzer (IO). Cl- ions were measured by DIONEX ICS-1100 (Wu QX, Han GL, Li FS Tang Y. Major element chemistry during the wet season in the upper Pearl River: A case study of the Nanpanjiang and Beipanjiang. Environ Chem 2015; 34: 1289–1296 (in Chinese with an English abstract).
(DOCX)
