# Correction: Sources and Dynamics of Inorganic Carbon within the Upper Reaches of the Xi River Basin, Southwest China

**DOI:** 10.1371/journal.pone.0169379

**Published:** 2016-12-28

**Authors:** Junyu Zou

There is an error in the second sentence of the Abstract. The correct sentence is: Carbonate dissolution and soil CO_2_ were regarded as the primary sources of DIC on the basis of δ^13^C_DIC_ values which varied along the Nanpan and Beipan Rivers, from −13.9‰ to −8.1‰.

There is an error in the sixth sentence of the Conclusions section: The correct sentence is: In addition, the involvement of sulfuric acid from coal related industries had a significant impact on the carbon evolution along the Beipan River.

Fig 3 appears incorrectly in the published article. Please see the correct Fig 3 and its caption below. Fig 3 is incorrect. The authors have provided a corrected version here.

**Fig 3:**
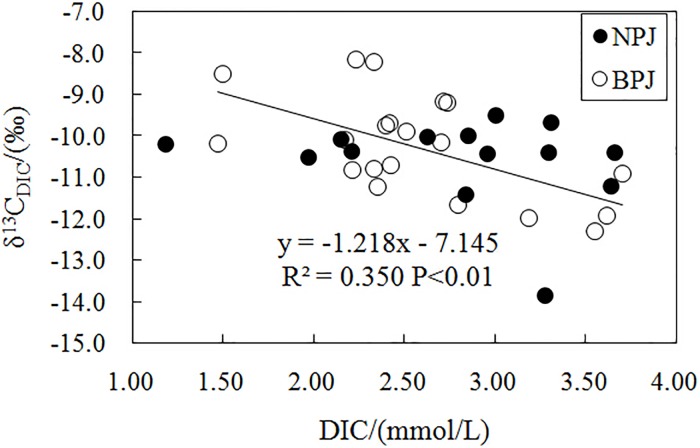
Correlation between DIC content and δ^13^C_DIC_. The trend line applies to data from the BPJ.
