# Supplementary material for: Sources and Dynamics of Inorganic Carbon within the Upper Reaches of the Xi River Basin, Southwest China
Source: PLoS One. 2016 Aug 11;11(8):e0160964. doi: 10.1371/journal.pone.0160964 (PMC4981298; doi:10.1371/journal.pone.0160964)
Supplement: S1 Table — BPJ and NPJ represent the mainstream and tributary of the Beipan River and the Nanpan River; “-”: undetected—quantity of solid sample was insufficient for measurement; “*”: no data—samples were not collected in the field; Distance refers to distance to the outlet of the basin (data was measured by Arcgis). DOC datasets cited from Zou (in review). DOC concentrations were determined on an Aurora 1030W TOC Analyzer (IO). Cl- ions were measured by DIONEX ICS-1100 (Wu QX, Han GL, Li FS Tang Y. Major element chemistry during the wet season in the upper Pearl River: A case study of the Nanpanjiang and Beipanjiang. Environ Chem 2015; 34: 1289–1296 (in Chinese with an English abstract). (DOCX) [file pone.0160964.s001.docx]

S1 Table

Sampling sites and geochemical index along the Nanpan and Beipan Rivers during the wet season.

| Sample | River | Latitude | Longitude | Distance | T | pH | DO | pCO2 | logpCO2 | SIc | DIC | PIC | δ^13^C_DIC_ | δ^13^C_PIC_ | Cl^-^ | DOC |
| --- | --- | --- | --- | --- | --- | --- | --- | --- | --- | --- | --- | --- | --- | --- | --- | --- |
|  |  |  |  | km | °C |  |  |  | μatm |  | mmol/l | mg/l | ‰ | ‰ | mmol/l | mg/l |
| BPJ-1 | Beipan River | N; 24º56.984' | E; 106º08.133' | 1 | 30.6 | 8.4 | 158.2% | 576 | -3.2 | 0.9 | 2.17 | - | -10.1 | - | 0.17 | 1.84 |
| BPJ-2 | Wangmo River | N; 25º09.598' | E; 106º05.519' | 66 | 26.6 | 8.1 | 110.8% | 1365 | -2.9 | 0.8 | 2.79 | - | -11.7 | - | 0.16 | 1.39 |
| BPJ-3 | Zhelou River | N; 25º02.840' | E; 105º55.993' | 42 | 30.4 | 8.4 | 119.9% | 705 | -3.2 | 1.0 | 2.51 | - | -9.9 | - | 0.12 | 1.25 |
| BPJ-4 | Beipan River | N; 25º04.399' | E; 105º57.121' | 41 | 23.7 | 8.1 | 105.3% | 1248 | -2.9 | 0.6 | 2.42 | - | -10.7 | - | 0.11 | 1.06 |
| BPJ-5 | Luofan River | N; 25º07.095' | E; 105º51.757' | 51 | 21.8 | 8.0 | 99.8% | 2266 | -2.6 | 0.7 | 3.55 | * | -12.3 | * | 0.17 | 1.05 |
| BPJ-6 | Groundwater | N; 25º06.965' | E; 105º51.617' | 52 | 22.1 | 7.4 | 58.3% | 10153 | -2.0 | 0.2 | 3.70 | * | -10.9 | * | 0.40 | 0.8 |
| BPJ-7 | Luofan River | N; 25º06.965' | E; 105º51.618' | 52 | 21.4 | 7.8 | 96.1% | 3296 | -2.5 | 0.6 | 3.61 | 0.17 | -11.9 | -1.8 | 0.17 | 1.03 |
| BPJ-8 | Baiceng River | N; 25º22.678' | E; 105º47.127' | 80 | 21.8 | 8.0 | 102.4% | 1515 | -2.8 | 0.5 | 2.33 | - | -10.8 | - | 0.11 | 3.68 |
| BPJ-9 | Baiceng River | N; 25º23.054' | E; 105º45.477' | 83 | 24.7 | 8.4 | 108.1% | 843 | -3.1 | 1.1 | 3.19 | - | -12.0 | -1.9 | 0.14 | 3.72 |
| BPJ-10 | Beipan River | N; 25º28.012' | E; 105º46.908' | 88 | 22.8 | 8.2 | 105.3% | 1037 | -3.0 | 0.6 | 2.35 | - | -11.2 | - | 0.11 | 3.83 |
| BPJ-11 | Beipan River | N; 25º31.932' | E; 105º45.989' | 95 | 27.0 | 8.3 | 121.6% | 737 | -3.1 | 0.9 | 2.33 | - | -8.2 | - | 0.14 | 4.43 |
| BPJ-12 | Dabang River | N; 25º52.383' | E; 105º40.367' | 140 | 20.5 | 8.1 | 102.5% | 1289 | -2.9 | 0.7 | 2.70 | 0.02 | -10.2 | -3.0 | 0.10 | 4.95 |
| BPJ-13 | Dabang River | N; 25º53.339' | E; 105º38.595' | 142 | 20.7 | 8.1 | 103.9% | 1220 | -2.9 | 0.6 | 2.39 | 3.63 | -9.8 | -2.3 | 0.10 | 4.12 |
| BPJ-14 | Baishui River | N; 26º00.774' | E; 105º40.083' | 155 | 21.1 | 8.2 | 103.6% | 1036 | -3.0 | 0.8 | 2.71 | 1.42 | -9.2 | -3.1 | 0.10 | 5.24 |
| BPJ-15 | Beipan River | N; 25º40.338' | E; 105º39.591' | 115 | 21.3 | 7.8 | 106.9% | 2570 | -2.6 | 0.4 | 2.74 | 3.36 | -9.2 | -0.8 | 0.09 | 3.84 |
| BPJ-16 | Zangke River | N; 26º10.138' | E; 105º11.266' | 196 | 28.5 | 8.4 | 128.0% | 379 | -3.4 | 0.7 | 1.49 | - | -8.5 | - | 0.11 | 6.5 |
| BPJ-17 | Baiche River | N; 26º26.503' | E; 105º04.567' | 244 | 18.0 | 8.2 | 107.4% | 746 | -3.1 | 0.7 | 2.21 | 3.80 | -10.8 | -2.4 | 0.08 | 3.79 |
| BPJ-18 | Balang River | N; 26º22.931' | E; 105º00.733' | 234 | 20.3 | 7.8 | 97.4% | 1606 | -2.8 | -0.1 | 1.47 | 0.01 | -10.2 | -1.5 | 0.07 | 2.71 |
| BPJ-19 | Dadu River | N; 26º17.388' | E; 104º42.789' | 253 | 20.2 | 8.2 | 106.5% | 906 | -3.0 | 0.6 | 2.23 | 6.87 | -8.1 | -2.0 | 0.11 | 3.64 |
| BPJ-20 | Qingshui River | N; 26º00.141' | E; 104º30.067' | 301 | 18.1 | 8.1 | 96.3% | 1121 | -3.0 | 0.5 | 2.42 | 5.52 | -9.7 | -1.7 | 0.10 | 3.64 |
| NPJ-1 | Nanpan River | N; 24º56.894' | E; 106º08.749' | 1 | 30.6 | 8.4 | 163.5% | 599 | -3.2 | 0.9 | 2.21 | - | -10.4 | - | 0.17 | 1.87 |
| NPJ-2 | Mabie River | N; 25º11.607' | E; 104º54.663' | 203 | 20.3 | 8.1 | 100.8% | 1105 | -3.0 | 0.5 | 2.15 | 3.55 | -10.1 | -1.5 | 0.16 | 3.16 |
| NPJ-3 | Wanfeng Lake | N; 24º52.129' | E; 105º01.755' | 170 | 27.1 | 8.1 | 102.0% | 980 | -3.0 | 0.5 | 1.97 | - | -10.5 | -3.4 | 0.17 | 7.35 |
| NPJ-4 | Duoyi River | N; 24º46.524' | E; 104º32.253' | 251 | 21.6 | 7.9 | 103.1% | 2313 | -2.6 | 0.4 | 2.62 | - | -10.0 | -1.9 | 0.17 | 3.83 |
| NPJ-5 | Qingshui River | N; 24º39.257' | E; 104º28.790' | 253 | 24.4 | 8.0 | 94.2% | 1918 | -2.7 | 0.7 | 3.00 | - | -9.5 | - | 0.06 | 5.42 |
| NPJ-6 | Nanpan River | N; 24º39.293' | E; 104º28.732' | 254 | 23.9 | 7.9 | 89.0% | 2606 | -2.6 | 0.5 | 2.95 | - | -10.4 | - | 0.27 | 7.28 |
| NPJ-7 | Dianxi River | N; 24º13.778' | E; 103º23.804' | 259 | 23.7 | 7.7 | 77.8% | 4637 | -2.3 | 0.5 | 3.64 | 1.51 | -11.2 | -5.6 | 0.40 | 8.13 |
| NPJ-8 | Huaxi River | N; 24º12.664' | E; 103º06.806' | 508 | 23.3 | 8.1 | 95.6% | 1455 | -2.8 | 0.7 | 2.84 | 2.08 | -11.4 | -1.8 | 0.56 | 11.04 |
| NPJ-9 | Nanpan River | N; 24º13.314' | E; 103º06.415' | 509 | 22.7 | 7.8 | 93.1% | 3051 | -2.5 | 0.6 | 3.27 | 4.41 | -13.9 | -2.5 | 0.44 | 10.41 |
| NPJ-10 | Nanpan River | N; 24º56.169' | E; 103º10.674' | 607 | 23.7 | 7.6 | 84.0% | 4486 | -2.3 | 0.2 | 2.85 | 0.19 | -10.0 | -3.5 | 0.39 | 12.4 |
| NPJ-11 | Nanpan River | N; 25º00.589' | E; 103º38.313' | 669 | 21.3 | 7.6 | 69.2% | 5006 | -2.3 | 0.3 | 3.29 | 2.13 | -10.4 | -4.7 | 0.48 | 14.69 |
| NPJ-12 | Nanpan River | N; 25º17.512' | E; 103º51.405' | 714 | 19.5 | 7.8 | 89.4% | 3693 | -2.4 | 0.5 | 3.65 | 1.83 | -10.4 | -7.1 | 0.22 | 8.24 |
| NPJ-13 | Nanpan River | N; 25º36.971' | E; 103º49.532' | 753 | 20.9 | 7.9 | 87.6% | 2467 | -2.6 | 0.5 | 3.31 | 0.77 | -9.7 | -5.8 | 0.34 | 11.25 |
| NPJ-14 | Nanpan River | N; 25º54.376' | E; 103º57.101' | 790 | 20.0 | 7.4 | 89.7% | 2697 | -2.6 | -0.9 | 1.18 | 1.78 | -10.2 | -9.1 | 0.03 | 8.34 |

BPJ and NPJ represent the mainstream and tributary of the Beipan River and the Nanpan River; “-”: undetected - quantity of solid sample was insufficient for measurement; “*”: no data - samples were not collected in the field; Distance refers to distance to the outlet of the basin (data was measured by Arcgis). DOC datasets cited from Zou (in review). DOC concentrations were determined on an Aurora 1030W TOC Analyzer (IO). Cl^-^ ions were measured by DIONEX ICS-1100 (Wu QX, Han GL, Li FS Tang Y. Major element chemistry during the wet season in the upper Pearl River: A case study of the Nanpanjiang and Beipanjiang. Environ Chem 2015; 34: 1289-1296 (in Chinese with an English abstract)).
